# Transglycosylation of gallic acid by using *Leuconostoc* glucansucrase and its characterization as a functional cosmetic agent

**DOI:** 10.1186/s13568-017-0523-x

**Published:** 2017-12-22

**Authors:** Seung-Hee Nam, Jeongjin Park, Woojin Jun, Doman Kim, Jin-A Ko, A. M. Abd El-Aty, Jin Young Choi, Do-Ik Kim, Kwang-Yeol Yang

**Affiliations:** 1 Institute of Agricultural Science and Technology, Chonnam National University, Gwangju, 61186 South Korea; 2 Division of Food and Nutrition, Research Institute for Human Ecology, Chonnam National University, Gwangju, 61186 Republic of Korea; 3 Department of International Agricultural Technology, Seoul National University, Gangwon do, 25354 Republic of Korea; 4 Microbiology and Functionality Research Group, World Institute of Kimchi, Gwangu, 6175 Republic of Korea; 5 Department of Pharmacology, Faculty of Veterinary Medicine, Cairo University, Giza, Egypt; 6 Department of Veterinary Pharmacology and Toxicology, College of Veterinary Medicine, Konkuk University, Seoul, 143-701 Republic of Korea; 7 Department of Chemistry and Research Institute of Life Science, Gyeongsang National University, Jinju, Republic of Korea; 8 Insect and Sericultural Research Institute, JARES, Jangsung, Republic of Korea; 9 Department of Plant Biotechnology, College of Agriculture and Life Sciences, Chonnam National University, Gwangju, 61186 Republic of Korea

**Keywords:** *Leuconostoc mesenteroides*, Dextransucrase, Gallic acid, Glucosylation, Whitening effect, Anti-aging effect

## Abstract

Gallic acid glycoside was enzymatically synthesized by using dextransucrase and sucrose from gallic acid. After purification by butanol partitioning and preparative HPLC, gallic acid glucoside was detected at *m/z* 355 (C_13_, H_16_, O_10_, Na)^+^ by matrix-assisted laser desorption ionization time-of-flight mass spectrometry. The yield of gallic acid glucoside was found to be 35.7% (114 mM) by response surface methodology using a reaction mixture of 319 mM gallic acid, 355 mM sucrose, and 930 mU/mL dextransucrase. The gallic acid glucoside obtained showed 31% higher anti-lipid peroxidation and stronger inhibition (*Ki* = 1.23 mM) against tyrosinase than that shown by gallic acid (*Ki* = 1.98 mM). In UVB-irradiated human fibroblast cells, gallic acid glucoside lowered matrix metalloproteinase-1 levels and increased the collagen content, which was indicative of a stronger anti-aging effect than that of gallic acid or arbutin. These results indicated that gallic acid glucoside is likely a superior cosmetic ingredient with skin-whitening and anti-aging functions.

**Electronic supplementary material:**

The online version of this article (10.1186/s13568-017-0523-x) contains supplementary material, which is available to authorized users.

## Introduction

Gallic acid (3,4,5-trihydroxylbenzoic acid) is abundant in leafy vegetables, fruits, nuts, such as gallnut, and different varieties of potatoes (Albishi et al. 2013; Atkinson et al. 2004; Chakraborty et al. 2009). In both the free form and as a part of hydrolyzable tannins, gallic acid is composed of three hydroxyl groups and one carboxyl group. Gallic acid forms intermolecular esters, such as digallic acid, trigallic acid, or cyclic ether-esters (Mämmelä et al. 2000). Gallic acid has been reported to elicit many biological effects, such as antioxidant, anticancer, antidiabetic, and anti-inflammatory activities (Abdelwahed et al. 2007; Noh and Lee 2011; Oboh et al. 2016; Pal et al. 2010; Yen et al. 2002). In particular, gallic acid derivatives, such as tannic acid and ellagic acid, inhibit melanogenesis or tyrosinase activity and serve as skin-whitening agents (Noh and Lee 2011; Panich et al. 2012).

Gallic acid is highly beneficial and has many industrial applications. It is inexpensive ($26–29/kg food-grade gallic acid), non-toxic to humans, and has no upper usage limit (Rajalakshmi et al. 2001; Yen et al. 2002). It has commonly been used as a standard for the determination of phenol content or as a starting source to synthesize the psychedelic alkaloid mescaline. In addition, gallic acid has been in demand as a functional cosmetic ingredient. However, the cosmetic applications of gallic acid are limited by its low solubility and bioavailability. The enzymatic modification of phenolic compounds by transglycosylation has gained interest as a mechanism to circumvent these difficulties, because such changes can improve the physiological function of polyphenolic compounds (Yoon and Robyt 2002; Moon et al. 2007a; Seo et al. 2009).

Dextransucrase catalyzes the synthesis of dextran from sucrose and transfers a glucose unit to other carbohydrates or phenolic compounds via glycosidic linkages (Moon et al. 2006; Robyt et al. 2008). The enzymatic transglycosylation by dextransucrase from *Leuconostoc mesenteroides* has been previously used for the modification of various bioactive compounds to improve their function or physical stability (Moon et al. 2007a; Seo et al. 2009). Our previous study showed that transglycosylated hydroquinone, a potential skin-whitening agent, inhibited tyrosinase or reduced melanin synthesis to a greater extent than hydroquinone (Kim et al. 2010).

In this study, we synthesized gallic acid glucoside from dextransucrase, purified the compound by preparative HPLC, and confirmed the synthesis by matrix-assisted laser desorption ionization time-of-flight mass spectrometry (MALDI-TOF MS). The optimum production conditions for gallic acid glucoside were determined by using response surface methodology. The functional properties of the gallic acid glucoside were studied to determine its potential as a cosmetic ingredient, including its antioxidant and anti-lipid peroxidation activities. The skin-whitening and anti-aging effects exerted by matrix metalloproteinase-1 (MMP-1) and collagen content were determined as well.

## Materials and methods

### Materials

Gallic acid, deuterium oxide (D_2_O), 1,1-diphenyl-2-picrylhydrazyl (DPPH), 3-(3,4-dihydroxylphenyl)-l-alanine (l-DOPA), mushroom tyrosinase, and β-arbutin were obtained from Sigma-Aldrich (St. Louis, MO, USA). All chemical reagents were commercially available and of analytical reagent grade.

### Enzyme preparation

Dextransucrase (EC 3.2.1.11) was obtained from *L. mesenteroides* B-512 FMCM (KCCM 11728P), which were cultured on LM medium with 2% (w/v) glucose, as previously described (Moon et al. 2007a). The fermented culture was harvested, centrifuged, and concentrated with 30 K hollow fibers (Millipore, Bedford, MA, USA). The enzyme activity was measured at 28 °C with 0.1 M sucrose in 20 mM sodium acetate (pH 5.2) for different reaction periods. The reactants were spotted on a thin-layer chromatography (TLC) silica gel 60 plate (Merck, Darmstadt, Germany) and developed twice in an acetonitrile–water (85:15, v/v) solution. The TLC plate was visualized by spraying with N-(1-naphthyl)-ethylenediamine-H_2_SO_4_ solution and heating at 121 °C for 10 min. The content of fructose released from sucrose was measured by the evaluation of its density using the NIH Image Program (http://rsb.info.nih.gov/nih-image) with a standard compound. One unit (U) was defined as the amount of enzyme that caused the release of 1 μmol of fructose per minute at 28 °C in 20 mM sodium acetate buffer (pH 5.2).

### Synthesis, purification, and identification of gallic acid glucoside

The reactants (1 L), which consisted of 325 mM gallic acid, 355 mM sucrose, and B-512 FMCM dextransucrase (0.55 mU/mL), were incubated in 20 mM sodium acetate (pH 5.2) at 28 °C for 6 h and boiled for 10 min to stop the enzyme action. Glucosylated gallic acid was confirmed by using TLC plate analysis (Merck, Darmstadt, Germany) at 25 °C. The reaction mixtures were placed on TLC plates and developed twice in the following solvent systems: (1) nitromethane/1-propanol/water (2:5:1.5, v/v/v) or (2) ethyl acetate/acetic acid/water (3:1:1, v/v/v) with gallic acid, fructose, and sucrose as the standard materials. Subsequently, the developed plate was visualized by spraying with N-(1-naphthyl)-ethylenediamine-H_2_SO_4_ solution and heating at 121 °C (Moon et al. 2007a) or UV exposure, as previously described (Seo et al. 2005).

The reaction mixture (1 L) was partitioned with n-butanol to obtain the modified gallic acid products from the upper layer. The modified products were further concentrated under vacuum to 50 mL by using a rotary evaporator (EYELA, Tokyo, Japan) at 47 °C. The sample was applied to a 4.0 × 75 cm silica gel column. After the removal of the remaining sugars with distilled water (total, 2.5 L; flow rate, 1 mL/min), gallic acid glucoside was extracted with 85% (v/v) acetonitrile in water. The compound was purified by high-pressure liquid chromatography (HPLC) under the following conditions: column TSK-GEL amide-80, 5 μm (Waters, Milford, MA, USA); 80% (v/v) acetonitrile in water mobile phase; 1.0 mL/min flow rate; RID-10A RI detector (Shimadzu, Tokyo, Japan).

Purified gallic acid glucoside (2 mg/mL) was mixed with 2,5-dihydroxybenzoic acid (1 mg/mL) in a ratio of 1:1 (v/v), loaded, and dried on a stainless-steel plate at 25 °C. The molecular mass of the sample was measured by MALDI-TOF (Voyager DE-STR, Applied Biosystems, Poster, CA, USA) in a linear mode with delayed extraction (75 laser shots) and an acceleration voltage of 65 kV.

### Optimization of gallic acid glucoside production

The influence of sucrose, enzyme, and gallic acid on the reaction was detected by using response surface methodology (RSM). The experimental data were applied via the response surface regression procedure with the following polynomial equation (Abe et al. 2000):

$$ \begin{aligned}Y &= \beta_{0} + \beta_{ 1} {\text{x}}_{ 1} + \beta_{ 2} {\text{x}}_{ 2} + \beta_{ 3} {\text{x}}_{ 3} \\ & \quad + \beta_{ 1 1} {{\text{x}}_{1}}^{ 2} + \beta_{ 2 2} {{\text{x}}_{ 2}}^{ 2} + \beta_{ 3 3} {{\text{x}}_{ 3}}^{ 2} \\ & \quad +  \beta_{ 1 2} {\text{x}}_{ 1} {\text{x}}_{ 2} + \beta_{ 1 3} {\text{x}}_{ 1} {\text{x}}_{ 3} + \beta_{ 2 3} {\text{x}}_{ 2} {\text{x}}_{ 3} . \end{aligned}$$

The regression and graphical analysis of the data were computed by Design-Expert 7.0.0 central composite design (CCD) RSM software (State-Ease, Minneapolis, MN, USA). The effects of separate parameters and interactions were analyzed by analysis of variance and the equation and model terms were analyzed by Fisher’s test for model significance. The fit quality for the model equation was indicated by the coefficient of determination (*R* ^*2*^) and an adjusted *R* ^*2*^. Preliminary experiments led to the selection of three factors (dextransucrase unit, sucrose, and gallic acid concentration) for the optimization of the production conditions of gallic acid glucoside, with the following values: dextransucrase from *L. mesenteroides*, 61–1238 mU/mL; sucrose, 10–700 mM; and gallic acid, 30–619 mM.

### Antioxidant activity

The antioxidant activities of gallic acid and gallic acid glucoside were detected by using a DPPH scavenging assay (Abe et al. 2000). Samples at each concentration (0.01–2.0 mM) were dissolved in ethanol, reacted with a 0.1 M DPPH reagent for 10 min at 25 °C, and monitored at 517 nm by using a microplate reader (Molecular Devices, Sunnyvale, CA, USA). The radical scavenging activity was expressed as the percentage inhibition of DPPH radical concentration against the reference compound of ascorbic acid. The IC_50_ value was designated as the concentration of sample that resulted in a 50% reduction in DPPH radicals.

### Anti-lipid peroxidation activity

The anti-lipid peroxidation effect was analyzed by ARA-L kit (ABCD GmbH, Berlin, Germany) with an HP-CLA chemiluminescence-measuring device (Tohoku Electronic Industrial, Tokyo, Japan). In accordance with the TIC (thermo-initiated chemiluminescence) method, the antioxidant species in the sample (gallic acid or gallic acid glucoside) were incubated with ample free radical-attached luminol to delay photon generation until the antioxidant species were consumed. The lag time(s) was proportional to the amount of antioxidant species in sample. Each sample (20 μL of 0.5, 1.0, and 5.0 mM) or α-tocopherol (20 μL of 10, 25, 50, and 100 μM/mL) was mixed with a reaction buffer (1.0 mL) at 37 °C, and the effects were measured (Sreejayan et al. 1997).

### Tyrosinase inhibition

The incubation mixture consisted of l-DOPA (0–5 mM, l-β-3,4-dihydroxyphenyl alanine) and mushroom tyrosinase (10 U/mL), in the presence or absence of gallic acid or gallic acid glucoside (0–10 mM) as the inhibitor. Ten units of mushroom tyrosinase were used to find the *Ki* value. The nature of the inhibition was determined by a Dixon plot of the relationship between the reciprocal of the velocity of the reaction and the concentration of the inhibitor (gallic acid or gallic acid glucoside at 0–10 mM) at various substrate concentrations (0.1, 0.5, 1.0, and 5.0 mM l-DOPA). After the mixture was incubated at 37 °C for 10 min, the absorbance was measured at 475 nm by using a microplate reader (Molecular Devices, Sunnyvale, CA, USA), which allowed the calculation of the tyrosinase inhibition (Kim et al. 2010).

### MMP-1 production and type 1 procollagen production by enzyme-linked immunosorbent assay (ELISA)

Human newborn foreskin fibroblast cells (HS68) were cultured in Dulbecco’s Modified Eagle’s Medium supplemented with 10% fetal bovine serum (Sigma-Aldrich, St. Louis, MO, USA) and 1% antibiotic–antimycotic (Sigma-Aldrich, St. Louis, MO, USA) in a humidified atmosphere with 5% CO_2_ at 37 °C. The HS68 cells (ATCC CRL 1635; Rockville, MD, USA) were subcultured in a 1:5 ratio for several passages until they reached 80–90% confluence (Ho et al. 2005; Watanabe et al. 2004). The serum-starved confluent cells were washed twice with phosphate-buffered saline (PBS), left in a thin layer of PBS, and exposed to UVB (100 mJ/cm^2^) from a UVB lamp (312 nm, Spectroline Model EB-160C, New York, NY). Immediately after irradiation, the cells were washed in a serum-free medium and the response was detected after incubation for 24 h. Prior to UVB irradiation, the cells were pretreated with arbutin (standard, Sigma-Aldrich, St. Louis, MO, USA), gallic acid, and gallic acid glucoside (10–100 μM/mL). The negative control (Control) comprised cells that did not receive UVB exposure and the positive control (UVB) comprised cells that received UVB exposure in the absence of an antioxidant compound (Ho et al. 2005; Watanabe et al. 2004). MMP-1 production was measured by using an ELISA kit (Merck & Co. Inc., Whitehouse Station, NJ, USA), as previously described (Ho et al. 2005). Type 1 procollagen content was measured by using a procollagen type I C-peptide ELISA kit (MK101, Takara, Tokyo, Japan), as previously described (Watanabe et al. 2004). Each sample was measured in triplicate.

## Results

### Synthesis, purification, and identification

**Fig. 1:**
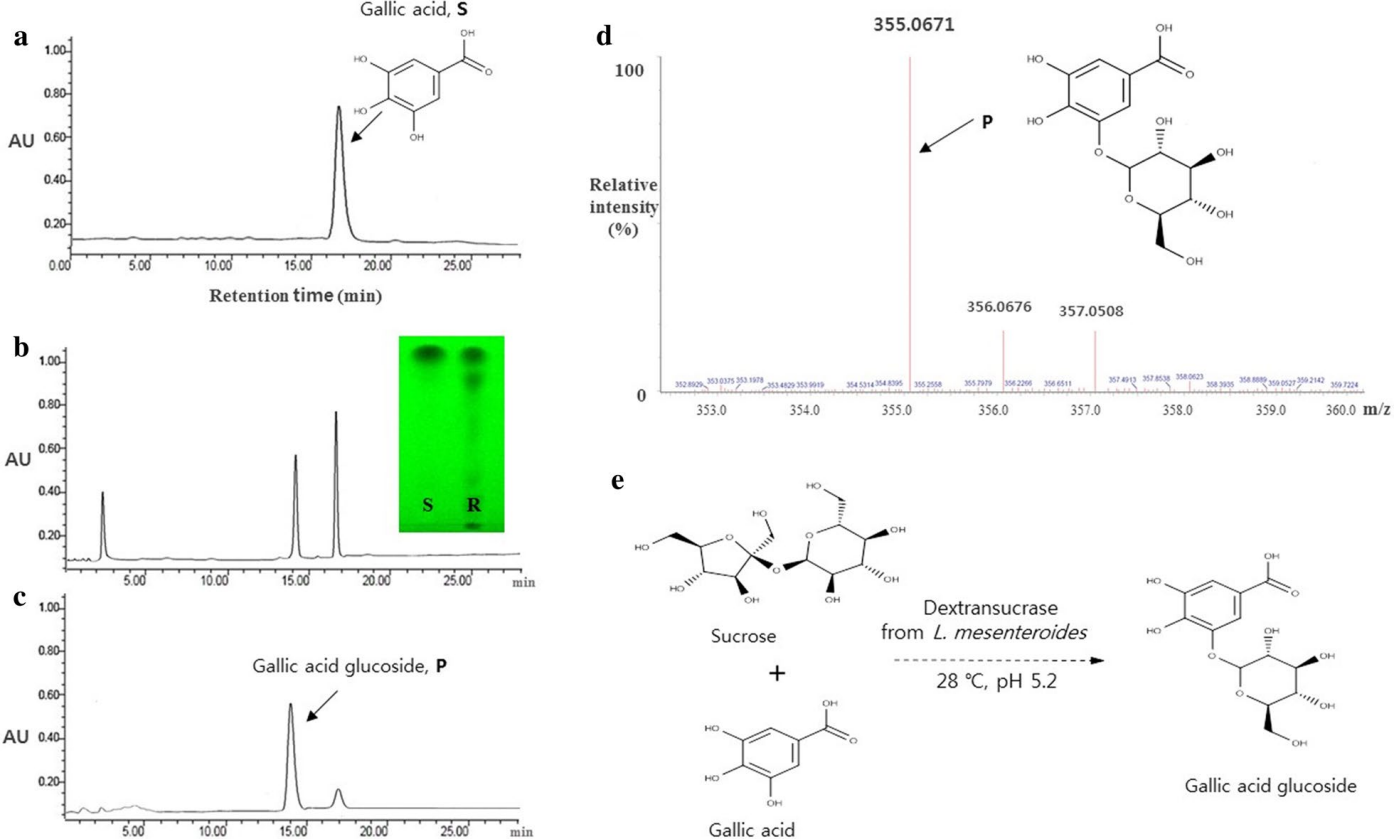
The schematic diagram of the reaction and HPLC chromatogram of gallic acid glucoside after preparative HPLC. Gallic acid standard (1 mg/mL) in ethanol (**a**), dextransucrase reaction digest for 6 h (**b**), purified gallic acid glucoside (**c**), MALDI-TOF MS spectrum of gallic acid glucoside (**d**), and the schematic diagram of the reaction (**e**). *S* gallic acid, *R* enzyme reaction mixture of gallic acid, and *P* final product. Column, TSK-GEL amide-80, 5 μm (Waters, Milford, MA, USA); mobile phase, acetonitrile/water = 80:20 (v/v); flow rate, 1.0 mL/min; room temperature; detection, RID-10A RI detector (Shimadzu)

Gallic acid glucoside was detected as a reaction product of dextransucrase with gallic acid and sucrose by TLC and HPLC (Fig. 1). The acceptor reaction mixture was purified by butanol partitioning, which removed the unreacted or hydrolyzed carbohydrates or enzymes present in the lower layer. The upper layer was enriched with gallic acid and the reaction product, gallic acid glucoside. Subsequently, gallic acid and gallic acid glucoside were separated by preparative HPLC using 80% (v/v) acetonitrile in water (Fig. 1c).

The purified gallic acid glucoside was obtained as a brownish-yellow powder with a purified yield of 12.0 g (62% of the total product synthesized). The number of glucose units attached to compounds was verified by using MALDI-TOF MS. The molecular ions of gallic acid glucoside were observed at *m/z* 355 (M+Na)^+^ (Fig. 1d and Additional file 1: Figure S1). The molecular weight of the compound was increased by adding a glucose moiety to the expected structure via a glycosidic linkage, as shown in Fig. 1d. Our studies revealed that dextransucrase from *L. mesenteroides* B-512 FMCM could synthesize α-1,3 glycoside linkage with phenolic compound like caffeic acid or astragalin (Nam et al. 2017; Kim et al. 2012).

### Optimum gallic acid glucoside synthesis

**Fig. 2:**
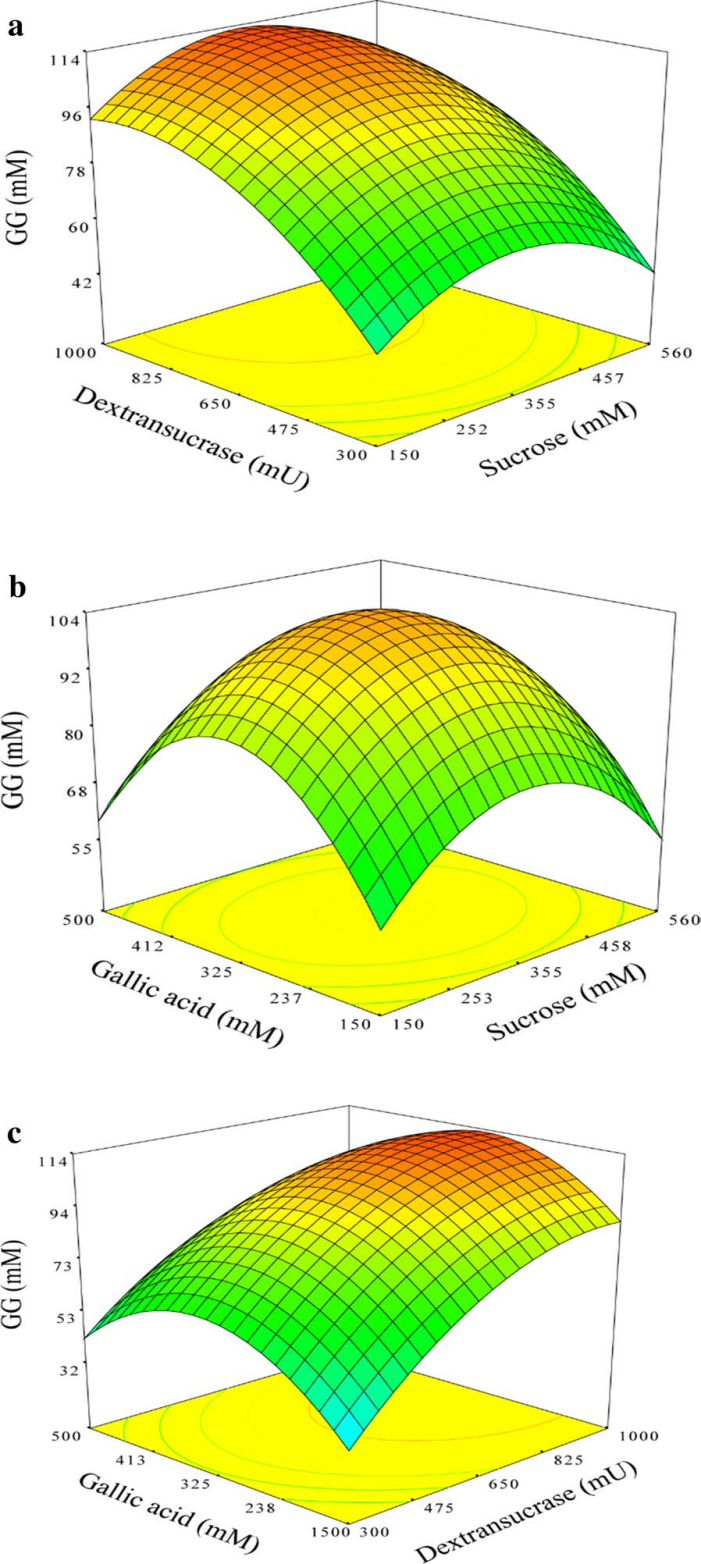
The response surface and contour plots of gallic acid glycoside (GG) production. The mutual interactions are shown between dextransucrase and sucrose (**a**), gallic acid and sucrose (**b**), and gallic acid and dextransucrase (**c**). The synthesis of gallic acid glycoside was optimized by dextransucrase from *L. mesenteroides* (61–1238 mU/mL), sucrose concentration (10–700 mM), and gallic acid concentration (30–619 mM)

The production of gallic acid glucoside was optimized by a CCD matrix by using the actual and predicted values, as shown in Table 1. The average amounts of gallic acid glucoside (mM) produced in 20 different experiments are presented in Table 1. The interactions of these variables were evaluated by RSM within the range from − 1.682 to + 1.682 (Fig. 2) and described by a second-order polynomial equation. The response (gallic acid glucoside production) was contained in the following regression equation:

**Table 1:** Central composite design matrix for the experimental design and predicted responses for gallic acid glucoside synthesis

Run no.	Coded levels			Gallic acid glucoside synthesis (mM)	
	X_1_	X_2_	X_3_	Actual	Predicted
1	150	300	150	29.0	13/9
2	560	300	150	22.4	10.9
3	150	1000	150	86.4	68.0
4	560	1000	150	80.9	69.0
5	150	300	500	43.5	24.5
6	560	300	500	32.5	19.9
7	150	1000	500	82.9	63.4
8	560	1000	500	78.7	62.8
9	10.2	650	325	21.6	49.5
10	699.8	650	325	30.7	46.5
11	355	61.4	325	0.2	19.8
12	355	1238.6	325	77.4	101.4
13	355	650	30.7	6.2	25.0
14	355	650	619.3	3.9	28.7
15	355	650	325	122.7	103.9
16	355	650	325	92.9	103.9
17	355	650	325	104.6	103.9
18	355	650	325	98.2	103.9
19	355	650	325	114.8	103.9
20	355	650	325	98.2	103.9

Y = − 158.9 + 0.32X_1_ + 0.24X_2_ + 0.63X_3_ − 0.00047X_1_^2^ − 0.00013X_2_^2^ − 0.00089X_3_^2^ + 0.0000139X_1_X_2_ − 0.000011X_1_X_3_ − 0.000062X_2_X_3_

$$ \begin {aligned} {\text{Y}} &= - 1 5 8. 9 + 0. 3 2 {\text{X}}_{ 1} + 0. 2 4 {\text{X}}_{ 2} + 0. 6 3 {\text{X}}_{ 3} \\ & \quad - 0.000 4 7 {{\text{X}}_{ 1}}^{ 2} - 0.000 1 3 {{\text{X}}_{ 2}}^{ 2} - 0.000 8 9 {{\text{X}}_{ 3}}^{ 2} \\ & \quad + 0.0000 1 3 9 {\text{X}}_{ 1} {\text{X}}_{ 2} - 0.0000 1 1 {\text{X}}_{ 1} {\text{X}}_{ 3} - 0.0000 6 2 {\text{X}}_{ 2} {\text{X}}_{ 3} \end {aligned}$$

where X_1_ was the sucrose concentration (mM), X_2_ was the dextransucrase unit (mU/mL), and X_3_ was the gallic acid concentration (mM). The results of the second-order response surface model fitting in ANOVA are presented in Table 1, Additional file 1; Tables S1 and S2. The goodness fit of the model was evaluated by the coefficient *R* ^*2*^. The multiple correlation coefficient was 0.82, which explained 82% of the variation in the response. As “adequate precision value” was indicative of the signal-to-noise ratio index, a value greater than 4 indicated the proper prerequisites for a good fitting model. The adequate precision value of this model was 4.95, which indicated that the model was capable of navigating the design space. The predicted value for gallic acid glucoside production was 104.0 mM; the observed data was 105.2 ± 11.4 mM at 355 mM sucrose, 650 mU/mL dextransucrase, and 325 mM gallic acid, which showed the similarity between the predicted and experimental syntheses of gallic acid glucoside (Table 1 and Fig. 2). The optimum yield for gallic acid glucoside was 114 mM or 35.7% by the reaction of 930 mU/mL dextransucrase with 319 mM gallic acid and 355 mM sucrose.

### Antioxidant activity and anti-lipid peroxidation

**Fig. 3:**
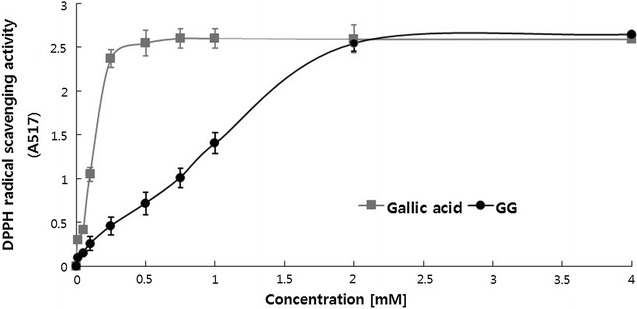
The DPPH radical-scavenging activity of gallic acid and gallic acid glucoside. The concentrations of 0, 0.01, 0.05, 0.1, 0.25, 0.5, 0.75, 1.0, and 2.0 mM were tested, and the reaction was monitored at 517 nm. The data are reported as mean ± SD of the three separate experiments

**Fig. 4:**
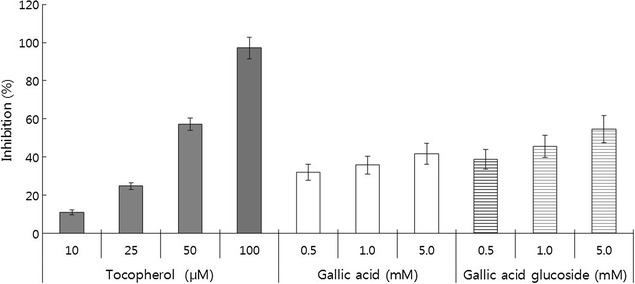
The inhibition of lipid peroxidation by gallic acid and gallic acid glucoside. The measurement was performed by using ARA-L kits obtained from ABCD GmbH and a chemiluminescence measuring device HP-CLA (Tohoku, Japan). α-Tocopherol (10, 25, 50, and 100 μM) was used as the control, and the extent of inhibition of lipid peroxidation by gallic acid and gallic acid glucoside (0.5, 1.0, and 5.0 mM) was determined

The antioxidant activities of gallic acid and its glucoside were determined by a DPPH scavenging assay (Fig. 3). The IC_50_ value of gallic acid glucoside was 0.94 mM, which was sevenfold higher than that of gallic acid (IC_50_ = 0.13 mM). In the terms of anti-peroxidation activity, gallic acid and gallic acid glucoside exhibited different magnitudes of inhibition of lipid peroxidation (Fig. 4). The use of α-tocopherol (10–100 μM) as a positive control resulted in a dose-dependent increase in chemiluminescent absorbance units (Y = 0.9671x + 2.8027, R^2^ = 0.9889). Gallic acid glucoside showed a significantly (*P* < 0.05) higher dose-dependent inhibitory effect (19–31%) than gallic acid (Fig. 4).

### Tyrosinase inhibition effects

**Fig. 5:**
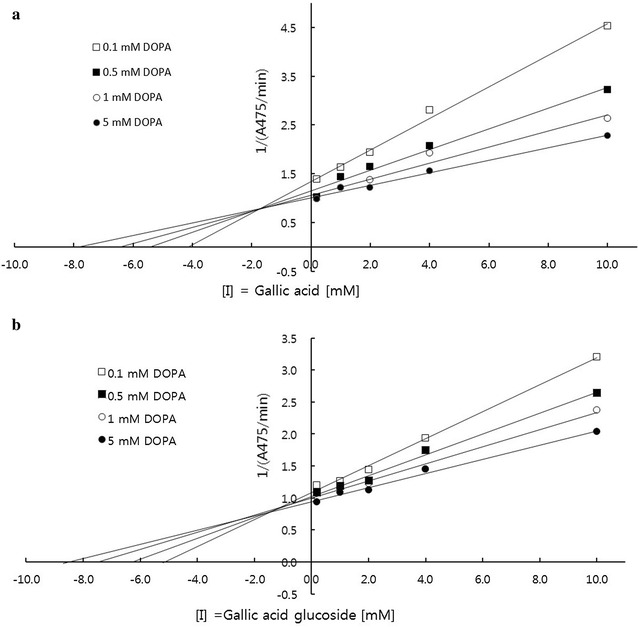
Dixon plots showing the reciprocal of the velocity (1/υ) of the mushroom tyrosinase reaction against the inhibitor 0–10 mM gallic acid (**a**) or gallic acid glucoside (**b**). Various substrate concentrations of l-DOPA (0.1, 0.5, 1.0, 5.0 mM) were applied to obtain the Dixon plots. A475 represents an increase in the absorbance at 475 nm

The kinetic studies of both gallic acid and its glucoside were performed by using tyrosinase-inhibitor complexes and different substrate concentrations (Fig. 5). The initial value was measured at various concentrations of substrate [l-DOPA ([S] = 0.1–5.0 mM)] and gallic acid and gallic acid glucoside (0–10 mM). The slope, s, and the vertical axis intercept, *i*, increased with an increase in gallic acid or gallic acid glucoside content (Fig. 5a and b). The corresponding reciprocal plot was linear and gallic acid and gallic acid glucoside was identified as a mixed noncompetitive inhibition type (Sugimoto et al. 2005). The secondary plots of the slopes and vertical axes intercepts from the Dixon plots against the gallic acid and gallic acid glucoside concentrations resulted in a straight line. The *Ki* values of gallic acid and its glucoside were calculated to be 1.98 and 1.23 mM, respectively; furthermore, the whitening effect of gallic acid glucoside was greater than that of gallic acid, which had a lower *Ki* value (Fig. 5).

### MMP-1 production and collagen content induced by UV irradiation

**Fig. 6:**
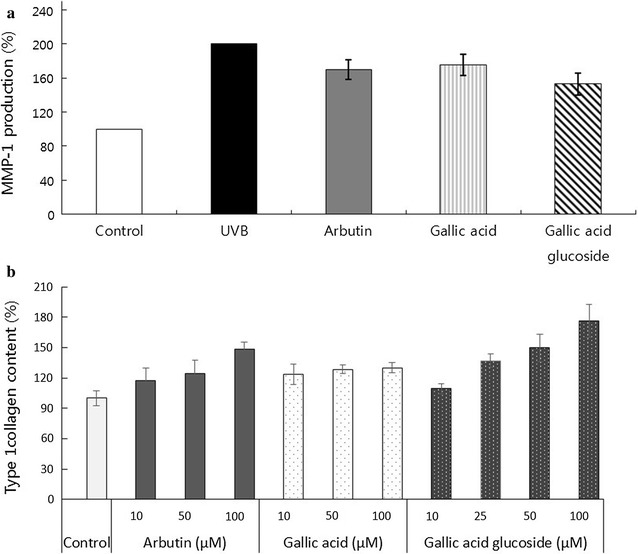
The anti-aging effect by UVB-induced MMP-1 production (**a**) and type I procollagen content (**b**) of arbutin, gallic acid, or gallic acid glucoside in human skin fibroblasts. The cells were pretreated with arbutin, gallic acid, or gallic acid glucoside (10 μM/mL or 10–100 μM/mL) before UVB irradiation (100 mJ/cm^2^) and harvested after 24 h. MMP-1 production and type 1 procollagen content were determined by using ELISA kits. Each bar represents the mean + SD (n = 5)

Matrix metalloproteinases (MMPs) play an important role in photo-aging through the facilitation of the degradation of extracellular matrix proteins. In particular, MMP-1 and interstitial collagenases have been reported to initiate the degradation of type I and III collagen levels after single or repeated UV exposure in human skin in vivo (Kim et al. 2011). The human-skin fibroblast cells were pretreated with arbutin, gallic acid, or gallic acid glucoside (10 μM/mL) prior to UVB irradiation. MMP-1 production was determined by ELISA kit and its content was normalized to the negative control (100%) or the positive control (200%). Gallic acid glucoside (153%) showed 22% stronger inhibition of MMP-1 than gallic acid (175%) (Fig. 6a). Arbutin showed a slightly higher inhibitory effect (170%) than gallic acid, with lower MMP-1 production. These results indicated that gallic acid glucoside displayed stronger inhibition of MMP-1 production either gallic acid or arbutin.

Collagen alteration has been reported as the primary explanation for skin aging and wrinkle formation (Jung et al. 2015). In addition to MMP-1, type I procollagen levels have also been measured in cells treated with gallic acid, gallic acid glucoside, or arbutin (10–100 μM/mL), as shown in Fig. 6b. When the collagen content was normalized to that of the positive control (100%), the collagen contents of three samples were positively correlated with their treatment concentrations (Fig. 6b). Among the samples, gallic acid glucoside resulted in higher collagen production than that induced by gallic acid or arbutin between 10 and 100 μM/mL. At 100 μM/mL, the treatment with gallic acid glucoside (176%) resulted in higher type I collagen production (46% or 27%), which was higher than that after treatment with gallic acid (130%) or arbutin (149%) (Fig. 6b).

## Discussion

Gallic acid glucoside is synthesized by the acceptor reaction of dextransucrase with gallic acid and sucrose via α-glycosidic linkage. A previous study has reported the synthesis of derivatives of gallic acid with a UDP-glucose substrate through glycosylation (β-linkage) using a glucosyltransferase extracted from oak leaves (Gross 1982).

The present data indicated that the attachment of a glucose or sugar moiety to gallic acid decreased its antioxidant activity, as shown in previous reports (Seo et al. 2009; Kim et al. 2010). The attachment of a galactose or glucose moiety (arbutin) to hydroquinone decreased its in vitro antioxidant activity tenfold in comparison with that of hydroquinone itself, irrespective of the attached sugar type (Seo et al. 2009; Kim et al. 2010). Gallic acid glucoside resulted in 19–31% higher anti-lipid peroxidation than gallic acid (Fig. 4). This result was consistent with a previous study that stated glycosylated caffeic acid markedly decreased the rate of auto-peroxidation of linoleic acid to a significantly greater extent than the aglycon compound (Nishimura et al. 1995).

Tyrosinase inhibitors have important applications as skin-whitening compounds in the cosmetic industry. Recently, new tyrosinase inhibitors, such as arbutin derivatives, have emerged as in-demand substances as new agents for depigmentation, cosmeceuticals, and skin-lightening compounds (Sugimoto et al. 2005). In a previous study, gallic acid was reported to show dose-dependent inhibition (IC_50_ = 3.5 mM) of l-DOPA oxidation catalyzed by mushroom tyrosinase (Kim 2007). This inhibition was 100 times stronger than that of kojic acid (Kim 2007; Nithitanakool et al. 2009). In a previous study, a gallic acid methyl derivative was shown to be a poorer inhibitor of the diphenolase activity of mushroom tyrosinase than gallic acid or kojic acid (Nithitanakool et al. 2009). Meanwhile, the glycosylated forms of gallic acid (pentagalloyl glucopyranose) isolated from the seed kernels of *Mangifera indica* (Nithitanakool et al. 2009) and the roots of *Paeonia suffruticosa* (Ding et al. 2009) have been reported to be 16 times stronger than gallic acid for the inhibition of mushroom tyrosinase in a non-competitive inhibition manner. The previous studies correlated well with this finding that glycosylated gallic acid (1.23 mM) showed a slightly stronger whitening effect with a lower *Ki* value than gallic acid itself (1.98 mM) by mixed noncompetitive inhibition, instead of competitive-type inhibition (Fig. 5).

Recently, it was reported that gallic acid can negatively modulate MMP-1 secretion and positively modulate elastin, type I procollagen, and transforming growth factor-β1 (Hwang et al. 2014). In addition, gallic acid can accelerate wound healing through the protection of skin cells and the promotion of cell migration under normal and hyperglucidic conditions (Yang et al. 2016). The present study focused on the role of glycosylated gallic acid in skin anti-aging. Glycosylated gallic acid was more efficient than gallic acid or arbutin in the prevention of aging through stronger MMP-1 inhibition and a greater induction of type 1 collagen (Fig. 6a and b). These results were consistent with our previous studies that showed that a glycosylated form of arbutin was a stronger inhibitor of UVB-induced MMP-1 production than arbutin (Moon et al. 2007b). The findings of this current study showed that gallic acid glucoside exerted a combined antityrosinase and anti-aging effect that was even stronger than that of gallic acid. As skin-whitening agents are associated with the dehydration of skin and the formation of wrinkles, gallic acid glucoside has demonstrated a strong potential to function as an additive in cosmetics for skin-whitening and anti-aging effects.

Collectively, this study showed that gallic acid glycoside could be synthesized from dextransucrase. It was purified and identified by MALDI-TOF MS. The optimum conditions of gallic acid glycoside were studied by RSM. In comparison with gallic acid or arbutin, gallic acid glycoside showed stronger anti-lipid peroxidation, anti-tyrosinase activity, and anti-aging function, with lower MMP-1 production but higher collagen content. Henceforth, gallic acid glycoside might be used as an applicable whitening and anti-aging ingredient in cosmetic products. Currently, significant efforts are in progress to increase the production yield of gallic acid glucoside using different enzymes and various solvents. In addition, the synthesis of gallic acid attached to 2–3 glucosides by dextransucrase is ongoing to confirm the relationship between the number of glucose attachments on gallic acid and its physical stability or function.

## Additional file

**Additional file 1: Figure S1.** MALDI ionization spectrum of gallic acid glucoside. **Table S1.** Independent variables, levels, and experimental codes used in response surface methodology (RSM). **Table S2.** ANOVA for RSM parameters fitted to second-order polynomial equations.

## Abbreviations

MALDI-TOF MS: matrix-assisted laser desorption ionization time-of-flight mass spectrometry
MMP-1: matrix metalloproteinase-1
TLC: thin-layer chromatography
HPLC: high-pressure liquid chromatography
RSM: response surface methodology
CCD: central composite design
TIC: thermo-initiated chemiluminescence
DPPH: 1,1-diphenyl-2-picrylhydrazyl
l-DOPA: 3-(3,4-dihydroxylphenyl)-l-alanine
MMPs: matrix metalloproteinases
