# Supplementary material for: Transglycosylation of gallic acid by using Leuconostoc glucansucrase and its characterization as a functional cosmetic agent
Source: AMB Express. 2017 Dec 22;7:224. doi: 10.1186/s13568-017-0523-x (PMC5741567; doi:10.1186/s13568-017-0523-x)
Supplement: Supplementary file 1 — Additional file 1: Figure S1. MALDI ionization spectrum of gallic acid glucoside. Table S1. Independent variables, levels, and experimental codes used in response surface methodology (RSM). Table S2. ANOVA for RSM parameters fitted to second-order polynomial equations. [file 13568_2017_523_MOESM1_ESM.docx]

**Supplemental data**

**Transglycosylation of gallic acid by using *Leuconostoc* glucansucrase and its characterization as a functional cosmetic agent**

Seung-Hee Nam^1^, Jeongjin Park^2^, Woojin Jun^2^*, Doman Kim^3^, Jin-A Ko^4^, A. M. Abd El-Aty^5,6^, Jin Young Choi^7^, Do-Ik Kim^8^, Kwang-Yeol Yang^9^*

^1^ Institute of Agricultural Science and Technology, Chonnam National University, Gwangju 61186, South Korea, email: namsh1000@hanmail.net

^2^ Division of Food and Nutrition, Research Institute for Human Ecology, Chonnam National University, Gwangju, 61186, Republic of Korea, email: pjj8425@hanmail.net

^3^ Department of International Agricultural Technology, Seoul National University, Gangwon do 25354, Republic of Korea, email: kimdm@snu.ac.kr

^4^ Microbiology and Functionality Research Group, World Institute of Kimchi, Gwangu 6175, Republic of Korea, email: jinarhdwn1@naver.com

^5^ Department of Pharmacology, Faculty of Veterinary Medicine, Cairo University, Giza, Egypt, email: abdelaty44@hotmail.com

^6^ Department of Veterinary Pharmacology and Toxicology, College of Veterinary Medicine, Konkuk University, Seoul 143-701, Republic of Korea, email: abdelaty44@hotmail.com

^7^ Department of Chemistry and Research Institute of Life Science, Gyeongsang National University, Jinju, Republic of Korea, email: cjy3541@naver.com

^8^ Insect and Sericultural Research Institute, JARES, Jangsung, Republic of Korea, email: doik961020@korea.kr

^9^ Department of Plant Biotechnology, College of Agriculture and Life Sciences, Chonnam National University, Gwangju, 61186, Republic of Korea, email: kyyang@jnu.ac.kr

*Corresponding authors

Kwang-Yeol Yang, Department of Plant Biotechnology, College of Agriculture and Life Sciences, Chonnam National University, Gwangju, 61186, Republic of Korea*.* Tel: +82-62-530-0207/ +82-010-4265-0308, Fax: +82-62-530-2069, E-mail: kyyang@jnu.ac.kr

Woojin Jun, Division of Food and Nutrition, Chonnam National University, Gwangju 61186, Republic of Korea. Tel: +82-62-530-1337 Fax: +82-62-530-2149, E-mail: wjjun@jnu.ac.kr

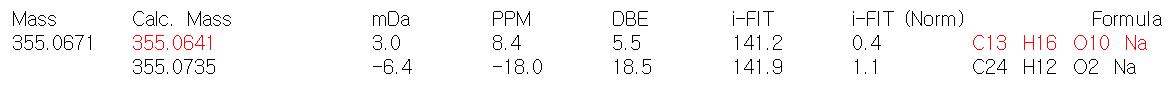


Expecting Size: 170.12+180+23-18 =**355.06**

**Formula=** C13 H16 O10 Na

**Fig. S1** MALDI ionization spectrum of gallic acid glucoside

**Table S1.** Independent variables, levels, and experimental codes used in response surface

methodology (RSM)^a^.

| Variables | Units | Symbol code | Levels | | | | |
| --- | --- | --- | --- | --- | --- | --- | --- |
|  |  |  | ‐ 1.682^b^ | ‐ 1 | 0 | + 1 | + 1.682^b^ |
| Sucrose | mM | x_1_ | 10.2 | 150 | 355 | 560 | 699.8 |
| Enzyme | (mU/mL) | x_2_ | 61.4 | 300 | 650 | 1000 | 1238.6 |
| Gallic acid | mM | x_3_ | 30.7 | 150 | 325 | 500 | 619.3 |

^a^ *Y* = β_0_ + β_1_x_1_ + β_2_x_2_ + β_3_x_3_ + β_11_x_1_^2^ + β_22_x_2_^2^ + β_33_x_3_^2^ + β_12_x_1_x_2_ + β_13_x_1_x_3_ + β_23_x_2_x_3_.

^b^ Based on program design value

**Table S2.** ANOVA for RSM parameters fitted to second-order polynomial equations.

| Source | Sum of squares | Degree of freedom | Mean square | *F*‐value | *P*‐value > *F* |
| --- | --- | --- | --- | --- | --- |
| Model | 24956.3 | 9 | 2772.9 | 4.95 | < 0.0099 |
| Residual | 5598.5 | 10 | 559.8 |  |  |
| Lack of fit | 4951.1 | 5 | 990.2 | 7.65 | 0.021 |
| Pure error | 647.4 | 5 | 129.5 |  |  |
| Cor Total | 30554.8 | 19 |  |  |  |

Standard Deviation = 23.66, R^2^ = 0.82, C.V. = 38.53, Adj‐R^2^ = 0.65
